# Meta-analysis of supplemental methionine effects on plasma amino acid concentrations in dairy cows

**DOI:** 10.3168/jdsc.2025-0804

**Published:** 2025-10-10

**Authors:** G.I. Zanton

**Affiliations:** USDA-Agricultural Research Service, US Dairy Forage Research Center, Madison, WI 53706

## Abstract

Increasing metabolizable Met (mMet) through feeding supplemental rumen-protected Met to dairy cows is a common component of balancing rations for Met. Changes in plasma Met (pMet) brought about by this practice may affect the metabolism of other AA, resulting in changes in the concentration of other plasma AA (pAA). The objective of this meta-analysis was to evaluate the changes in pAA when lactating dairy cows were provided supplemental mMet. Literature studies were identified that fed cows a control diet and the control diet supplemented with Met as either rumen-protected Met or through infusion to increase pMet. There were 41 studies feeding 60 control and 78 Met treatments that met the selection criteria and entered into the final analysis. Responses entering the meta-analysis were calculated as Met-supplemented cow pAA − control cow pAA and analyzed as weighted mean differences or standardized weighted mean differences where the weighting term was the inverse variance and robust variance estimation was conducted to account for the hierarchical structure of the data. Regression was also conducted where pAA were regressed against pMet weighted by the inverse variance and including the random effect of study and experiment-within-study. Mean pMet in control cows was 19.9 µ*M*, which increased with supplementation by 11.3 µ*M*. Sulfur-containing pAA Cys and homocysteine as well as Lys were increased with increasing pMet whereas Gln, Glu, His, Ile, Leu, Tyr, and Val decreased with increasing pMet. The pAA Arg, Gly, Orn, Ser, Tau, and Trp responded curvilinearly to pMet with the predicted response in Arg, Orn, Tau, and Trp reaching a peak and Gly and Ser a nadir at intermediate concentrations of pMet. Milk protein yield increased in association with greater pMet, but responses to supplemental Met appeared to be limited by the reduction of other pAA, as higher responses to pMet were observed at higher levels of dietary CP. These results imply that concentration changes in pAA should be considered during diet formulation when feeding supplemental mMet to support increased milk protein production.

## Highlights

• Increased plasma Met (pMet) resulted in changes in several plasma AA (pAA).
• Plasma concentrations of several essential AA and nonessential AA were inversely related to pMet.
• Sulfur AA in plasma were increased with increasing pMet.
• Changes in pAA should be considered when increasing metabolizable Met.

## Graphical Abstract

**Figure:**
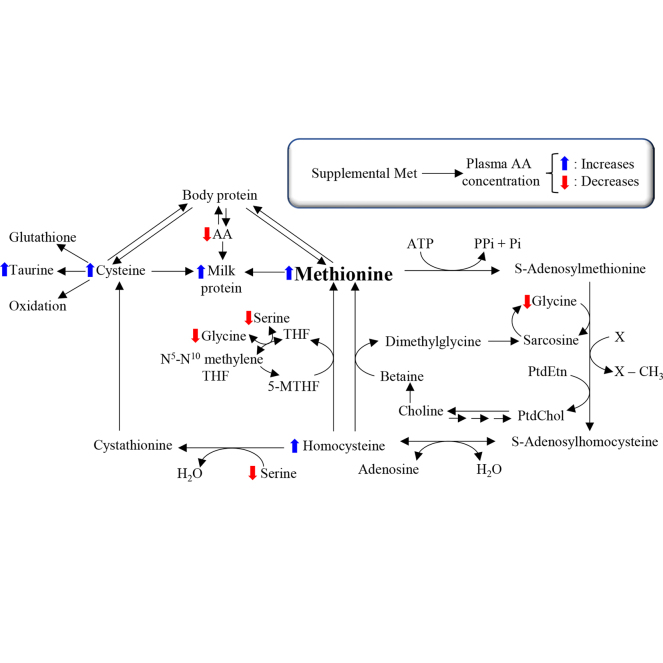
**Summary:** Supplementing rumen-protected methionine (Met) is a common practice on dairy farms. The hypothesis of this study was that cows provided supplemental Met would have altered plasma amino acid (AA) concentrations indicative of AA metabolism in response to altered plasma Met. This question was addressed through a meta analysis of peer-reviewed studies that supplemented lactating cows with rumen-protected Met and evaluated plasma AA concentrations. Increased plasma Met resulted in reduced plasma concentrations of several AA required for milk protein synthesis and greater reductions in AA involved in methyl-donor reactions. Sulfur containing AA increased as plasma Met increased. Supplementing Met resulted in increased milk protein secretion, which was comparable in magnitude to the reduction of the concentration of several plasma AA. Milk protein production responses to supplemental Met were limited by the reduction of other plasma AA, and greater performance response to Met could be achieved by considering this response during diet formulation. PPi = diphosphate; Pi = inorganic phosphate; PtdEtn = phosphatidylethanolamine; PtdChol = phosphatidylcholine; THF = tetrahydrofolate; MTHF = methylenetetrahydrofolate.

Formulating lactating dairy cow diets for ideal AA composition is an important component of precision nutrition to optimize production, nitrogen use efficiency, and feed costs (Haque et al., 2012). Variation in feed and rumen microbial AA composition should be balanced to optimize supply and profile of absorbed AA (Hanigan et al., 2024), otherwise a suboptimal ration AA profile can result in reduced productivity (Letelier et al., 2022). Altering plasma AA concentration (**pAA**) through dietary formulation, rumen-protected (**RP**) AA supplementation (Patton et al., 2015), or casein infusion (Martineau et al., 2017) leading to increased metabolizable AA supply has been associated with increased milk protein yield (**MPY**). Feeding supplemental metabolizable Met (**mMet**) has become an accepted practice on dairy farms due to the production performance benefits that are often observed (Zanton et al., 2014). However, altering absorption of a single AA such as Met may create an imbalance in pAA profile that may result in unintended physiological or production effects (Weekes et al., 2006). Considering the extensive database and use of RP Met in many studies, the literature was summarized through meta-analysis to investigate the interrelationships among AA in cows receiving supplemental Met. The objective of this analysis was to determine the response of pAA concentrations to altered plasma Met (**pMet**) achieved through supplemental mMet. The hypothesis was that cows with increased pMet would have altered pAA indicative of AA metabolism.

Literature was searched according to PRISMA guidelines (Page et al., 2021) to identify studies in which cows were fed a diet either unsupplemented (control) or supplemented with Met as either RP Met or through postruminal (or intravenous) infusion. The source of additional Met (dietary or infusion) is only considered the means to increase pMet in this analysis. The search was conducted in PubMed (https://pubmed.ncbi.nlm.nih.gov/), Scopus (https://www.scopus.com/), and Web of Science (webofscience.com) databases using the following search terms: rumen AND protected OR escape AND methionine AND cow AND blood OR plasma. All available dates were included, but the search was restricted to peer-reviewed articles published in English (last searched on January 22, 2025). There were 158 records identified from PubMed, 2,139 from Scopus, 683 from Web of Science, and 9 from post hoc searching citations. After duplicates were removed, 2,370 records were screened from the title, abstract, tables, and figures. After screening, 120 reports were assessed for eligibility according to the following criteria: (1) within a study, Met was the only AA changed versus a control (studies in which complete feedstuffs or other AA [i.e., Lys] changes confounding predicted AA and Met flow were excluded); (2) studies had to report multiple pAA; (3) Met sources must be replicated in >5 publications; (4) chemical analogs of Met were excluded; (5) no challenge treatments or conditions during pAA reporting phase (including heat stress, mammary biopsy, and so on); (6) pulse dose studies were excluded; and (7) only postpartum pAA results were used (although supplementation may have begun prepartum). If an experiment or treatment set within a study met these selection criteria, that was retained for further analysis. Finally, 41 studies published between 1972 to 2023 met the selection criteria with 61 experiments (control diets not supplemented with Met), where the reference list and raw dataset are included in the supplemental data file (see Notes). In addition to pAA and their SE, experimental unit replicates and experimental design, source and amount of supplemental mMet, dietary CP and NDF concentration, breed and production, and BUN were recorded. If milk CP (g/d) was reported, it was converted to milk true protein by multiplying by a fixed conversion factor of 95.1% (NASEM, 2021).

Responses in Met supplementation were calculated as Met-supplemented cow pAA − control cow pAA (**δAA**, where a δ prefix here and subsequently indicates a response to supplemental Met in that variable) or as standardized mean differences (**SMD**) as δAA/SD_AA_ (**_smd_AA**). If cell mean values from a factorial experiment were reported in a study, then each factor basal diet was considered the control for each Met-supplemented diet. These were then also considered as unique experiments within a study (for example, in the study of Overton et al., 1998, one control and treatments were ground shelled corn, no RP Met vs. ground shelled corn, with RP Met, which is one experiment; corn gluten feed, no RP Met vs. corn gluten feed, with RP Met was considered a second comparison and experiment). Productive performance responses were calculated and analyzed the same as δAA and _smd_AA. Before statistical analysis, outlying observations of pAA and δAA were removed when the |studentized residuals| ≥ 4 (183/2,550 pAA); all pAA were removed if pMet was determined to be an outlier based on this criterion (Smartamine M in Blum et al., 1999, and 40 g Met in Varvikko et al., 1999). A threshold of 4 was used as a conservative threshold, as these data points represent calculated means from a group of cows and should have limited influence from outliers. After removing outlying observations, there were 60 control and 78 Met-supplemented treatments for pMet, in which 53% of studies were designed as changeovers that included 40.5% of the total control cow-periods. Meta-analysis of mean differences and SMD were conducted in the metafor package (Viechtbauer, 2010) of R with the clubSandwich option for cluster robust variance estimation to account for the hierarchical structure of the data (Pustejovsky and Tipton, 2022). Data were weighted using the inverse of the variance with study and experiment-within-study as random effects, where the within study effect size correlation as assumed to be rho = 0.8 (Pustejovsky and Tipton, 2022). The greatest weights were trimmed to the weight of the 90th percentile to avoid overweighting some studies. Dietary continuous moderators (CP and NDF) were also explored for effects on δMPY. Weighted, univariate correlations among δAA were also evaluated using the Corr procedure of SAS and visualized by a heatmap.

Because there was a wide range of Met supplementation levels, resulting in a wide range of pMet concentration, pAA concentrations were also regressed against pMet concentration weighted by the inverse variance using the Mixed procedure of SAS. The regression approach included pMet centered around the control mean pMet concentration (19.9 µ*M*), linear and quadratic parameter estimates (where the quadratic parameter estimate was excluded if *P* > 0.05), and the random effect of study and experiment-within-study (St-Pierre, 2001). If either random effect was estimated at 0, only the random effect for experiment was included to maintain parsimony. The fit of this regression was assessed by calculating R^2^ for the fixed effects (conditional on the presence of the random effects of study), raw square root of the mean squared error (**RMSE**), and RMSE scaled to the intercept when pMet = 19.9. Results with *P* < 0.05 are considered significant.

Across the 60 control observations (560 cow-periods), mean pMet was 19.9 µ*M* (95% CI: 18.50, 21.29], which is comparable (20.4–24.0 µ*M*) to other summarized literature (Patton et al., 2015; Martineau et al., 2019; Letelier et al., 2022). Control cows produced 29.1 kg/d milk (95% CI: 27.18, 31.05); 943 g MPY/d (95% CI: 882, 1,004); 1,108 g fat/d (95% CI: 1,036, 1,181); were 66 DIM (95% CI: 47, 85) at the start of the study; and consumed diets with 15.6% CP (95% CI: 15.13%, 16.05%) and 33.9% NDF (95% CI: 32.25%, 35.62%). Studies in this meta-analysis used 3 sources of Met: infused Met (12 studies, 18 experiments), dietary RP Met in the form of Smartamine (16 studies, 22 experiments), and Mepron (13 studies, 20 experiments). Overall δMet was 11.3 µ*M* (95% CI: 8.80, 13.78; Figure 1A) resulting in mean pMet of 33.2 µ*M* (95% CI: 30.94, 35.37) for Met-supplemented cows. Similar to previous meta-analyses (Zanton et al., 2014; Patton et al., 2015), increases in δMPY and concentration (21.1 g/d [95% CI: 8.59, 33.64]; 0.08% [95% CI: 0.054%, 0.102%]; Figure 1A) were observed whereas δfat (4.4 g/d [95% CI: −15.78, 24.61]; 0.04% [95% CI: −0.020%, 0.093%]) were not different. Milk yield, DMI, and BUN responses were unaffected by Met supplementation.

**Figure 1:**
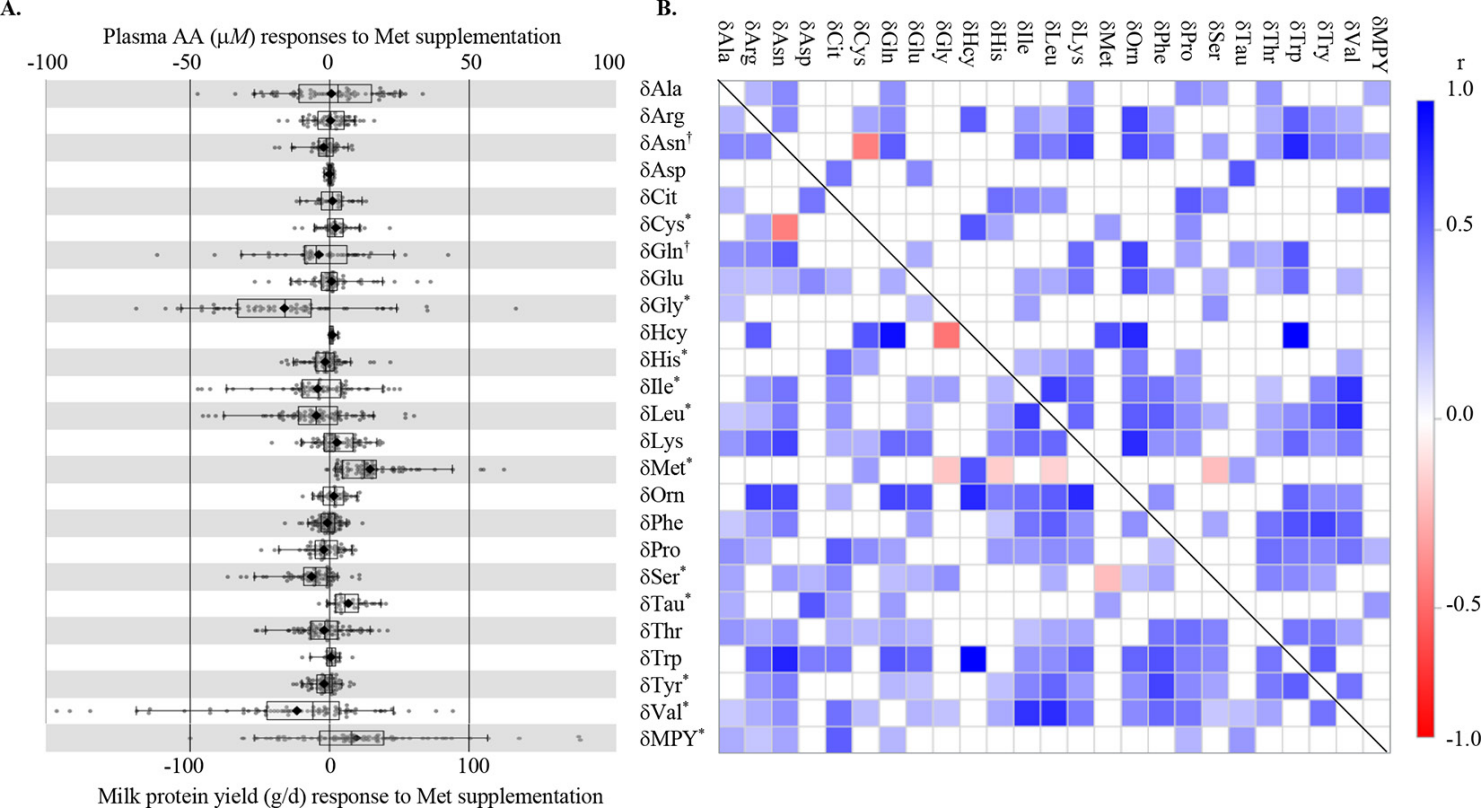
Effects of supplementing lactating dairy cows with metabolizable Met on plasma AA concentration responses (δAA = supplemented − control; µ*M*) or milk protein yield responses (δMPY, g). (A) Distributions of δAA or δMPY with overlaid box-and-whisker plot with whiskers representing 90% of the data, the box representing 50% of the data, interior line is the median, the diamond is the mean. *Indicates mean responses differ from 0 with *P* ≤ 0.05 and † indicates 0.05 < *P* ≤ 0.10. (B) Correlations among δAA and δMPY, where *P* ≤ 0.15 for correlations below the diagonal and *P* ≤ 0.05 above the diagonal.

Supplemental mMet increased Cys, Met, and Tau whereas Gly, His, Ile, Leu, Ser, Tyr, and Val decreased (Figure 1A). Likewise, average SMD for MPY (**_smd_MPY**) was +0.256 (95% CI: 0.0925, 0.4198), in contrast _smd_Met was +1.43 (95% CI: 1.131, 1.722). Group 1 AA (His, Met, Phe+Tyr, and Trp) are characteristically removed from arterial circulation and directly secreted into milk protein in a 1:1 uptake:output, whereas Group 2 AA (Arg, Ile, Leu, Lys, Val) typically have an uptake:output exceeding 1 (Mepham, 1982). In this analysis, most pAA changed similarly to the extent expected if being used from plasma for additional MPY produced in response to supplemental mMet, although whether this is due to removal or metabolism in the mammary gland or other tissues cannot be determined from the results of this study. A significant decline occurred for Gly, His, Leu, Ser, Tyr, and Val (ranging from SMD of −0.139 for Gly to −0.387 for Ser). These differences could not be distinguished statistically from decreased pAA concentration resulting from removal for MPY synthesis, which was determined as SMD for AA overlapping with 95% CI for negative MPY: reflecting secretion. Most other _smd_AA also did not differ from _smd_MPY but were also not different from 0 (i.e., no response to pMet). With the exceptions of δMet × δSer and δAsn × δCys, most significant univariate correlations were positive (Figure 1B). This indicates that pAA changes within studies generally responded similarly to treatment even though the responses were unrelated to δMet.

There were 16 AA associated with changes in pMet concentration in regression analysis (Table 1; Figure 2). Observed regression responses were predominantly linear with respect to pMet, although several pAA responded curvilinearly to pMet. Sulfur-containing AA Cys and Hcy as well as Lys increased with increasing pMet, whereas Arg, Orn, Tau, and Trp increased over the lower range of pMet up to the predicted peak concentrations at intermediate pMet (39.28, 39.13, 71.86, and 41.9 µ*M* pMet, respectively). The EAA His, Ile, Leu, and Val declined with increasing pMet. This could be due to increased pMet resulting in greater mammary gland clearance of pAA to support additional MPY as can occur for additional MP (Omphalius et al., 2019), reduced absorption of other dietary AA through antagonism by luminal Met (Moe et al., 1987), or increased extramammary tissue utilization (Patton et al., 2015). The largest reduction in pAA to increased pMet was Gly followed by Val and Ser although Gly and Ser responded curvilinearly and was at nadir at an intermediate pMet (62.38 and 59.70 µ*M* pMet, respectively). The greater reductions in Gly and Ser concentration are likely related to the integration of Met, Gly, and Ser in methyl donor metabolism whereas Ser is also involved in transsulfuration through the conversion of Hcy to Cys. When transition dairy cows were provided supplemental folic acid, which is involved in remethylation of Hcy to Met, Duplessis et al. (2017) found that plasma Cys and Hcy increased, whereas Gly concentrations declined. This was hypothesized to be due to the need to dispose of the excess 1-C units, while maintaining pMet. Similarly, if pMet increases through supplementation, there would likely need to be an increased consumption of Gly, and potentially Ser, due to the need to dispose of excess 1-C units and S through transmethylation and transsulfuration, respectively. Random effect of study was highly influential with study typically accounting for >90% of variance not explained by the fixed effects of the model. This could be due to factors not included in the statistical models such as dietary components, genetics, or management, and potentially, analytical methods for pAA. Ultimately, this analysis reveals that supplementing with Met resulted in altered pAA and generally reduced plasma EAA.

**Table 1:** Plasma AA (pAA) regression parameter estimates to increasing plasma Met in lactating dairy cows^1^

Group^5^	pAA	n^2^			Average (µ*M*) (pMet = 19.9)		Linear coefficient to (pMet − 19.9)			Quadratic coefficient^3^ to (pMet − 19.9)			Fit^4^			δ_11.3µ_*_M_*, µ*M*^6^
		Cont	Trt	Obs	β_o_	SE	β_1_	SE	*P*<	β_2_	SE	*P <*	R^2^	$\hat{\sigma_{e}}$	$\hat{\frac{\sigma_{e}}{\beta_{o}}}$	
S	Cys	30	38	587	33	7.6	0.25	0.029	<0.001				0.768	1.931	5.90	2.85
	Hcy	11	11	253	4	0.4	0.10	0.011	<0.001				0.891	1.915	48.51	1.18
	Tau	25	36	532	38	2.0	0.53	0.078	<0.001	−0.0051	0.00221	0.026	0.768	1.712	4.47	5.33
																
Methyl	Gly	44	61	957	331	12.5	−1.30	0.299	<0.001	0.0153	0.00658	0.024	0.417	1.284	0.39	−12.77
	Ser	47	64	983	87	4.5	−0.43	0.088	<0.001	0.0054	0.00202	0.010	0.499	1.325	1.53	−4.13
	Thr	59	76	1,217	96	5.2	−0.06	0.047	0.194				0.031	1.129	1.18	−0.70
																
AAA	His	54	65	1,118	48	2.7	−0.08	0.026	0.002				0.202	1.248	2.59	−0.92
	Phe	58	75	1,205	47	1.6	−0.03	0.020	0.103				0.056	1.254	2.69	−0.38
	Tyr	47	63	936	44	2.6	−0.06	0.023	0.015				0.120	1.105	2.49	−0.64
	Trp	18	24	499	46	6.2	0.11	0.048	0.031	−0.0025	0.00103	0.027	0.232	1.124	2.45	0.93
																
Lys	Lys	55	72	1,179	76	2.6	0.10	0.049	0.043				0.100	1.253	1.65	1.14
																
Urea	Arg	49	60	1,050	71	3.2	0.31	0.089	0.001	−0.0080	0.00224	0.001	0.410	1.144	1.61	2.46
Cycle	Cit	21	29	448	80	3.7	−0.04	0.038	0.303				0.050	1.142	1.42	−0.45
	Orn	25	39	536	48	4.0	0.15	0.053	0.009	−0.0031	0.00117	0.013	0.193	0.885	1.86	1.25
	Pro	43	57	880	90	4.1	−0.04	0.041	0.333				0.029	0.896	1.00	−0.45
																
BCAA	Ile	58	72	192	117	4.5	−0.13	0.058	0.029				0.089	1.316	1.12	−1.46
	Leu	57	74	1,193	145	6.8	−0.25	0.060	<0.001				0.276	1.152	0.80	−2.85
	Val	58	75	1,185	238	10.3	−0.38	0.087	<0.001				0.288	1.202	0.51	−4.32
																
NEAA	Ala	49	66	1,023	233	6.9	0.03	0.089	0.704				0.003	1.256	0.54	0.38
	Asn	29	38	692	48	3.3	−0.05	0.027	0.072				0.132	1.019	2.14	−0.57
	Asp	33	37	730	7	0.8	−0.01	0.007	0.210				0.054	1.185	17.14	−0.10
	Gln	34	43	758	193	12.8	−0.40	0.120	0.002				0.284	1.119	0.58	−4.50
	Glu	46	60	974	87	12.1	−0.05	0.024	0.049				0.107	0.950	1.09	−0.54
																
MPY, g/d		60	78	1,226	925	39.6	0.70	0.239	0.004				0.169	694	75	7.93

1 Control cows (n = 60 control diets, 560 cow-periods) had a mean pMet of 19.9 µ*M* (95% CI: 19.10, 22.70) and supplemented cows had a mean pMet response of 11.3 µ*M* (95% CI: 8.80, 13.78; 78 treatment diets, 666 treatment cow-periods). Equations were fit with the pAA regressed against pMet centered at the control mean pMet concentration (i.e., x = pMet − 19.9).

2 Number of control treatments (Cont), supplemented treatments (Trt), and total number of control and treatment cow-periods (Obs, when part of a longitudinal study, 1 cow-period = 1 cow) for each pAA, respectively.

3 Only included when β_2_ was significant at *P* < 0.05.

4 R^2^ is conditional on the presence of the random effect of study, $\hat{\sigma_{e}}$ is the root mean squared error, and $\hat{\frac{\sigma_{e}}{\beta_{o}}}$ is the root mean squared error scaled to β_o_.

5 S = sulfur-containing AA; Methyl = AA involved in methyl-donor reactions; AAA = AA with an aromatic ring; urea cycle = AA involved in the urea cycle; BCAA = branched-chain amino acids.

6 δ_11.3µ_*_M_* = pAA response when pMet changes at the mean increase of 11.3 µ*M*.

**Figure 2:**
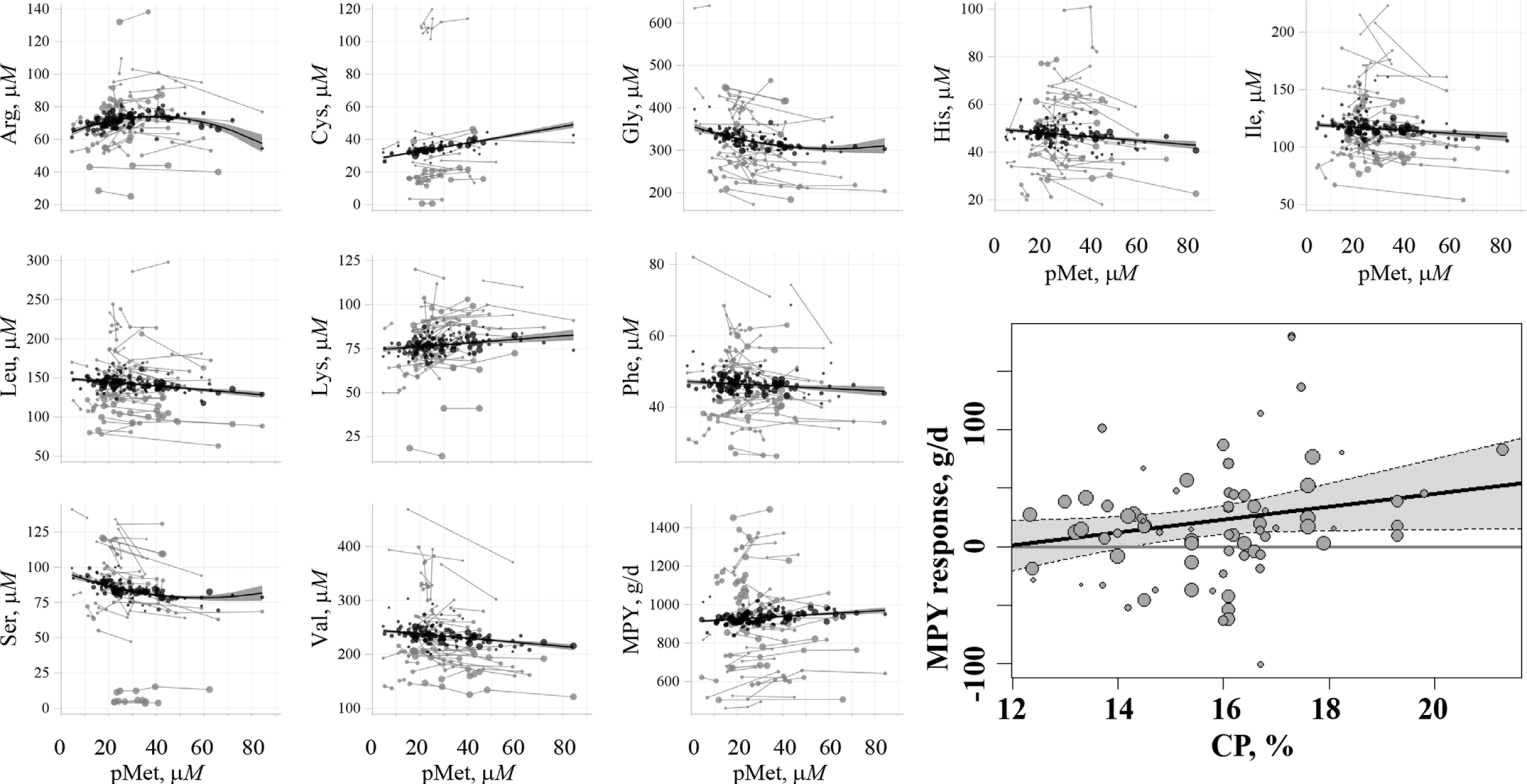
Select responses in proteinaceous pAA (µ*M*) or milk protein yield (MPY, g/d) to changing concentrations of pMet (µ*M*) or the mean difference response in MPY to dietary CP (% of DM) for cows provided increasing amount of metabolizable Met. Gray circles size indicates weight of the raw data in the analysis, with lines connecting circles coming from the same study. Black circles are adjusted results from the mixed model analysis with the best fit line and 95% CI. Note differences in y-axis scales for different AA.

Due to the number of factors that were unavailable from published manuscripts, and resulting high number of assumptions that would have been required for dietary modeling, in this study, reported chemical factors associated with metabolizable AA (CP) was used as a proxy in the covariate analysis. Response of MPY was affected by pMet where each µ*M* increase in pMet resulted in 0.70 g/d in MPY (Table 1). Additionally, the magnitude of the MPY response was affected by the level of CP in the basal diet (where * signifies *P* < 0.05; pseudo-R^2^ = 0.098, bias-corrected and accelerated bootstrap 95% CI: 0.000–0.290; Figure 2) where

$$\delta MPY,\frac{g}{d}=-65\left(\pm 37.5\right)+5.5\left(\pm 2.44^{*}\right)\times CP.$$

There were no effects detected for NDF levels on the δMPY in this analysis. This analysis revealed that MPY depended on the basal diet to which mMet was supplemented, with the largest responses to pMet occurring at higher levels of CP, although considerable variability in the MPY response to Met was not explained by CP alone.

The relationship of MPY with pMet and dietary CP is consistent with the declining pAA concentrations observed with increasing pMet, where lower CP may have placed a limitation on the extent of MPY response that was available from mMet supplementation. This could potentially result from EAA concentrations reduced beyond the capacity of the mammary gland to compensate through altered extraction efficiency especially at higher energy levels (Omphalius et al., 2019) or an accentuation of an AA profile imbalance in these conditions resulting in reduced productivity (Letelier et al., 2022). These results are consistent with a previous meta-analysis that showed higher responses in milk yield to supplemental Met when control diets contained higher levels of CP (Robinson, 2010) and earlier work showing higher milk protein content responses from adding mMet and metabolizable Lys to diets with higher CP compared with lower CP (Rulquin, 1992), although MPY had increased for both CP groups. The study by Socha et al. (2005) also showed an interaction between dietary CP and mMet supplementation for milk protein content although this was not detected in MPY. There were no interactions between dietary CP and Met supplementation in MPY in several other studies (Leonardi et al., 2003; Broderick et al., 2008, 2009) although in all of these studies supplemental mMet did not affect MPY yield at either CP level, the low CP level was ≥15.8, and it is unknown the extent to which pMet was affected by the Met treatments, as pAA were not reported. Formulating diets for metabolizable AA through manipulation of multiple dietary AA (Haque et al., 2012) or RUP sources and optimized energy content may allow for both reduced CP and enhanced responsiveness to the addition of supplemental Met. In particular, supplementing with ingredients with greater levels of metabolizable AA that were shown to decline with Met supplementation in this study may be beneficial but would need to be evaluated with additional research.

The conclusions from this meta-analysis are that the predominant response to increased pMet was a decline in the concentration of several EAA and some NEAA, especially those involved in Met metabolism. It is implied that improved production responsiveness to increased pMet may occur in diets formulated to attenuate the reduction of pAA.
